# Supplementary material for: Design, Synthesis, and Antisickling Investigation of a Thiazolidine Prodrug of TD-7 That Prolongs the Duration of Action of Antisickling Aromatic Aldehyde
Source: Pharmaceutics. 2023 Oct 28;15(11):2547. doi: 10.3390/pharmaceutics15112547 (PMC10675597; doi:10.3390/pharmaceutics15112547)
Supplement: Supplementary file 1 [file pharmaceutics-15-02547-s001.zip › pharmaceutics-2658446-supplementary.pdf]

Design, Synthesis, and Antisickling Investigation of a Thiazolidine Prodrug of TD-7 That Prolongs the Duration of Action of Antisickling Aromatic Aldehyde

Rana T. Alhashimi [1], Tarek A. Ahmed [2,3,*], Lamya Alghanem [4], Piyusha P. Pagare [4], Boshi Huang [4], Mohini S. Ghatge [4], Abdelsattar M. Omar [1], Osheiza Abdulmalik [5], Yan Zhang [4] and Martin K. Safo [4]

[1] Department of Pharmaceutical Chemistry, Faculty of Pharmacy, King Abdulaziz University, Alsulaymanyah, Jeddah 21589, Saudi Arabia; ralhashimi@kau.edu.sa (R.T.A.); asmansour@kau.edu.sa (A.M.O.)
[2] Department of Pharmaceutics, Faculty of Pharmacy, King Abdulaziz University, Alsulaymanyah,
Jeddah 21589, Saudi Arabia
[3] Centre for Artificial Intelligence in Precision Medicines, King Abdulaziz University, Alsulaymanyah,
Jeddah 21589, Saudi Arabia
[4] Department of Medicinal Chemistry and The Institute for Structural Biology, Drug Discovery and
Development, School of Pharmacy, Virginia Commonwealth University, Richmond, VA 23298, USA;
alghaniml@vcu.edu (L.A.); pagarepp@vcu.edu (P.P.P.); bhuang2@vcu.edu (B.H.); msghatge@vcu.edu (M.S.G.); yzhang2@vcu.edu (Y.Z.); msafo@vcu.edu (M.K.S.)
[5] Division of Hematology, The Children's Hospital of Philadelphia, Philadelphia, PA 19104, USA;
abdulmalik@email.chop.edu
* Correspondence: tabdelnapy@kau.edu.sa

Table S1. Stability studies of TD-7 and Pro-7 at different pHs

	pH 2.0			pH 7.4			pH 8.0		
Time (hr)	Pro-7	TD-7	DMSO	Pro-7	TD-7	DMSO	Pro-7	TD-7	DMSO
0	1.22 ± 0.4	3.23 ± 0.08	0.002	0.14 ± 0.01	3.05 ± 0.10	0.0005	0.14 ± 0.00	1.82 ± 0.05	0.007
1	3.16 ± 0.2	3.02 ± 0.00	0.0009	0.16 ± 0.00	2.97 ± 0.01	0.002	0.12 ± 0.00	1.79 ± 0.00	0.0002
2	3.17 ± 0.09	3.03 ± 0.04	0.001	0.17 ± 0.00	2.95 ± 0.03	0.0001	0.15 ± 0.00	1.73 ± 0.01	0.001
3	3.36 ± 0.03	2.97 ± 0.04	0.001	0.09 ± 0.09	3.09 ± 0.06	0.0004	0.17 ± 0.00	1.73 ± 0.01	0.002
6	3.27 ± 0.02	2.98 ± 0.01	0.002	0.22 ± 0.00	2.92 ± 0.02	0.003	0.24 ± 0.01	1.71 ± 0.01	0.003
24	3.05 ± 0.00	2.93 ± 0.01	0.003	0.49 ± 0.00	2.98 ± 0.07	0.001	0.64 ± 0.16	1.73 ± 0.02	0.0002
28	ND	ND	ND	0.55 ± 0.00	2.90 ± 0.04	0.001	1.00 ± 0.05	1.70 ± 0.02	0.0003
48	ND	ND	ND	0.98 ± 0.02	3.00 ± 0.05	0.0006	1.33 ± 0.32	1.68 ± 0.03	0.005
56	ND	ND	ND	1.10 ± 0.03	2.98 ± 0.08	0.0003	ND	ND	ND
72	ND	ND	ND	1.77 ± 0.07	2.92 ± 0.05	0.003	1.89 ± 0.04	1.73 ± 0.03	0.005
96	ND	ND	ND	2.59 ± 0.03	2.95 ± 0.03	0.006	1.86 ± 0.01	1.68 ± 0.04	0.0001

(2-(3-((6-(hydroxymethyl)pyridin-2-yl)methoxy)-5-methoxyphenyl)thiazolidin-4-yl)propan-1-one, **(Pro-7)**

^{1}HNMR

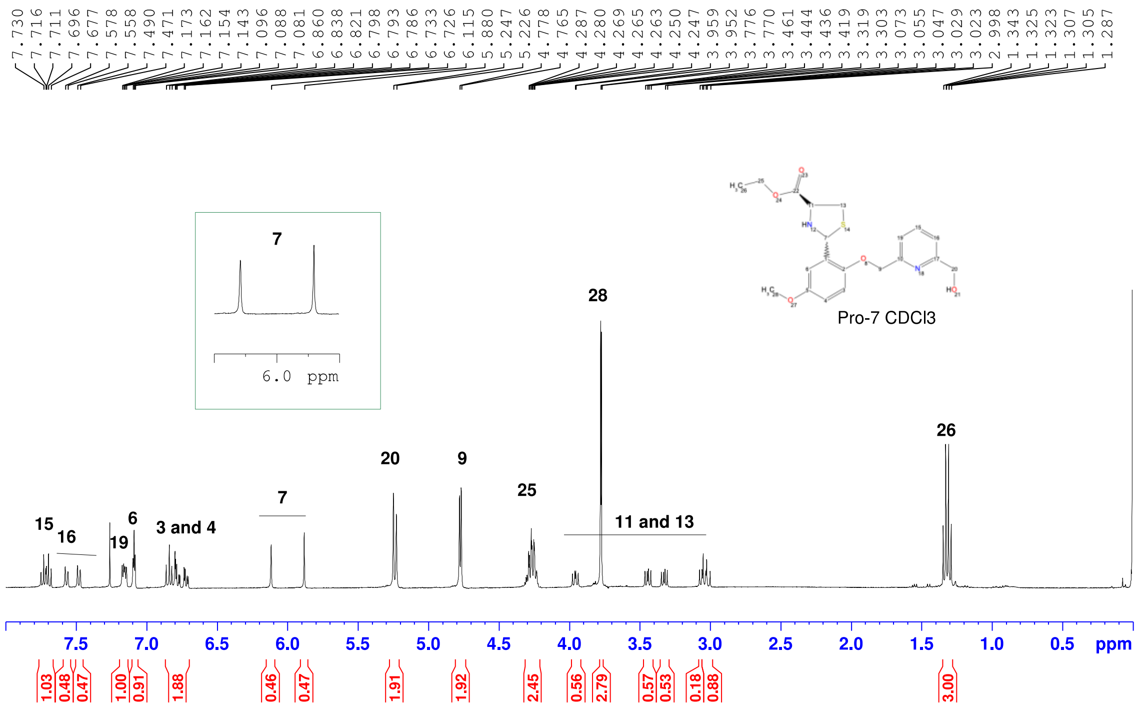

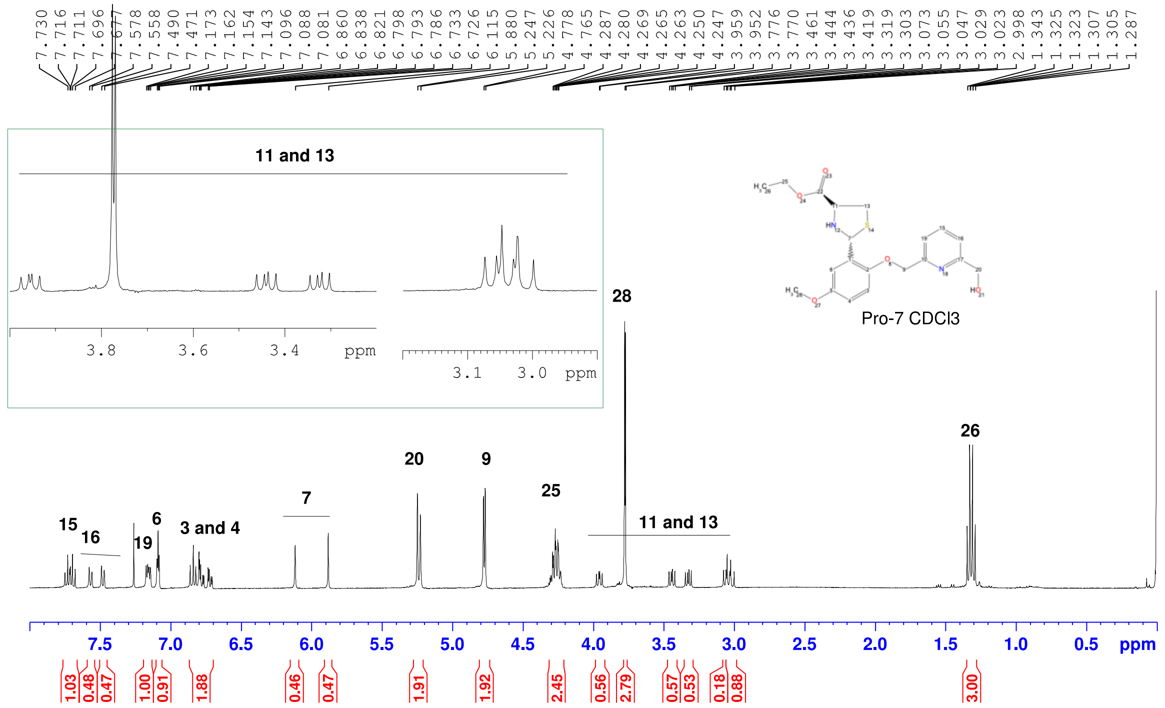
7.730 7.716 7.711 7.696 7.677 7.578 7.490 7.471 7.173 7.162 7.154 7.143 7.096 7.088 7.081 6.860 6.838 6.821 6.798 6.793 6.786 6.733 6.726 6.115 5.880 5.247 5.226 4.778 4.765 4.287 4.280 4.269 4.265 4.263 4.250 4.247 3.959 3.952 3.776 3.770 3.461 3.444 3.436 3.419 3.319 3.303 3.073 3.055 3.047 3.029 3.023 2.998 1.343 1.325 1.323 1.307 1.305 1.287
11 and 13
3.8 3.6 3.4 ppm
3.1 3.0 ppm
Pro-7 CDCl3
28
26
20
9
25
7
15
16
6
19
3 and 4
11 and 13
7.5 7.0 6.5 6.0 5.5 5.0 4.5 4.0 3.5 3.0 2.5 2.0 1.5 1.0 0.5 ppm
1.03 0.48 0.47 1.00 0.91 1.88 0.46 0.47 1.91 1.92 2.45 0.56 2.79 0.57 0.53 0.18 0.88 3.00

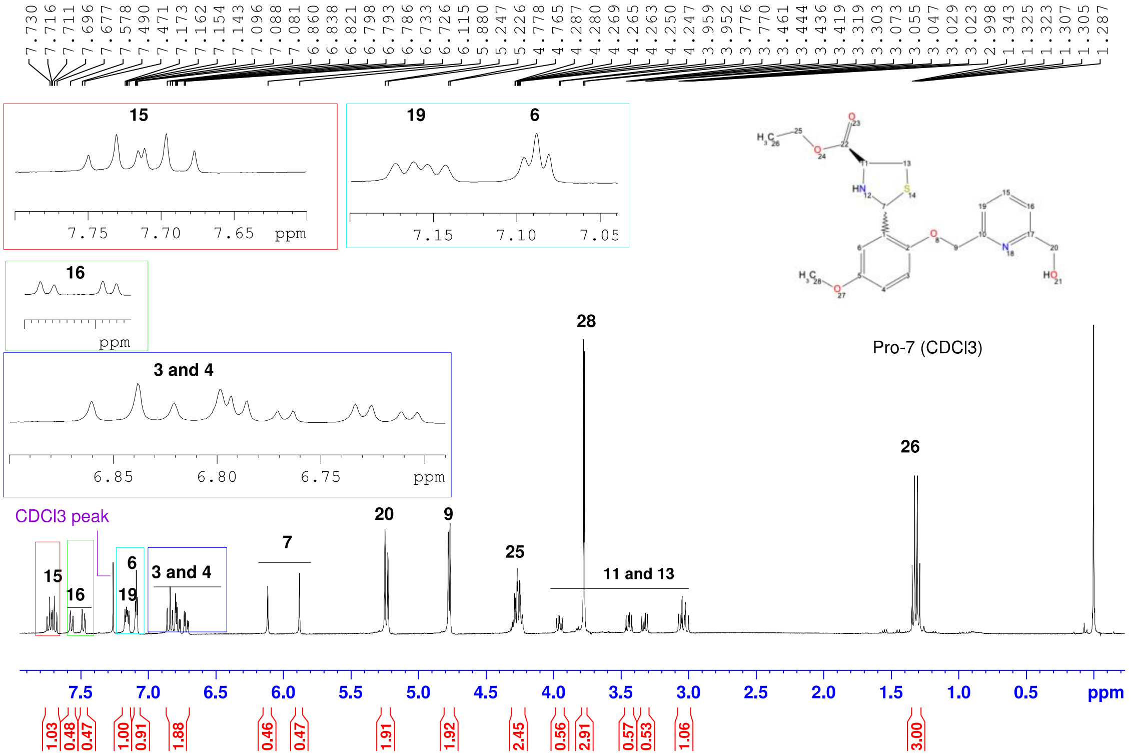
7.730 7.716 7.711 7.696 7.677 7.578 7.490 7.471 7.173 7.162 7.154 7.143 7.096 7.088 7.081 6.860 6.838 6.821 6.798 6.793 6.786 6.733 6.726 6.115 5.880 5.247 5.226 4.778 4.765 4.287 4.280 4.269 4.265 4.263 4.250 4.247 3.959 3.952 3.776 3.770 3.461 3.444 3.436 3.419 3.319 3.303 3.073 3.055 3.047 3.029 3.023 2.998 1.343 1.325 1.323 1.307 1.305 1.287
15
7.75 7.70 7.65 ppm
19
6
7.15 7.10 7.05
16
ppm
3 and 4
6.85 6.80 6.75 ppm
Pro-7 (CDCl3)
CDCl3 peak
28
26
20
9
25
7
15
16
6
19
3 and 4
11 and 13
7.5 7.0 6.5 6.0 5.5 5.0 4.5 4.0 3.5 3.0 2.5 2.0 1.5 1.0 0.5 ppm
1.03 0.48 0.47 1.00 0.91 1.88 0.46 0.47 1.91 1.92 2.45 0.56 2.91 0.57 0.53 1.06 3.00

^{13}CNMR

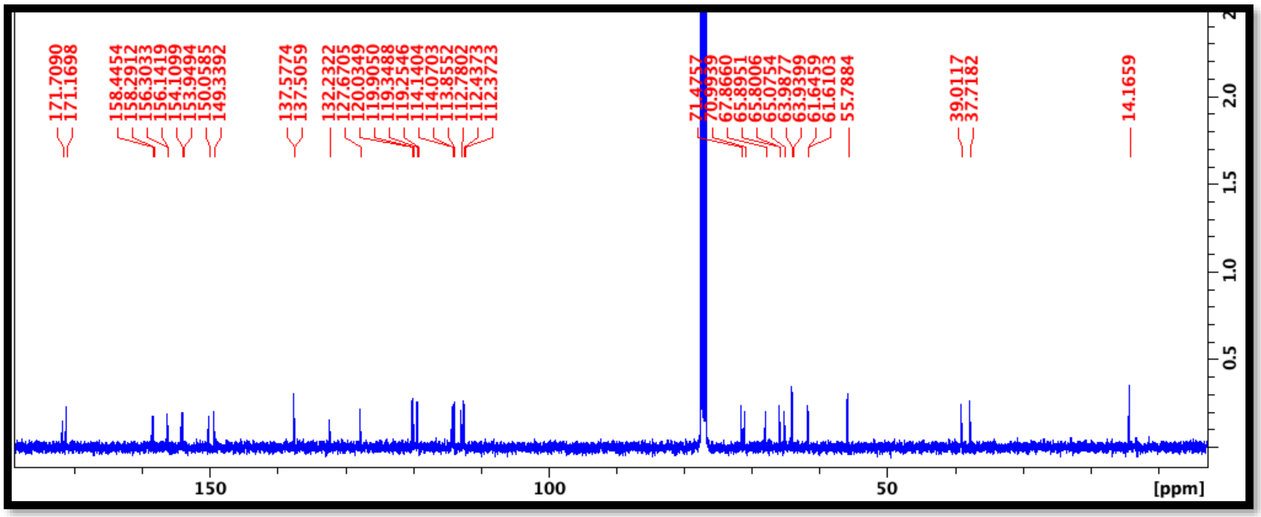

171.7090
171.1698
158.4454
158.2912
156.3033
156.1419
154.1099
153.9494
150.0585
149.3392
137.5774
137.5059
132.2322
127.6705
120.0349
119.9050
119.3488
119.2546
114.1403
114.0703
113.8552
112.7802
112.4373
112.3723
71.4757
67.8660
65.8951
65.8006
65.0754
63.9877
63.9399
61.6459
61.6103
55.7884
39.0117
37.7182
14.1659
150
100
50
[ppm]

HRMS

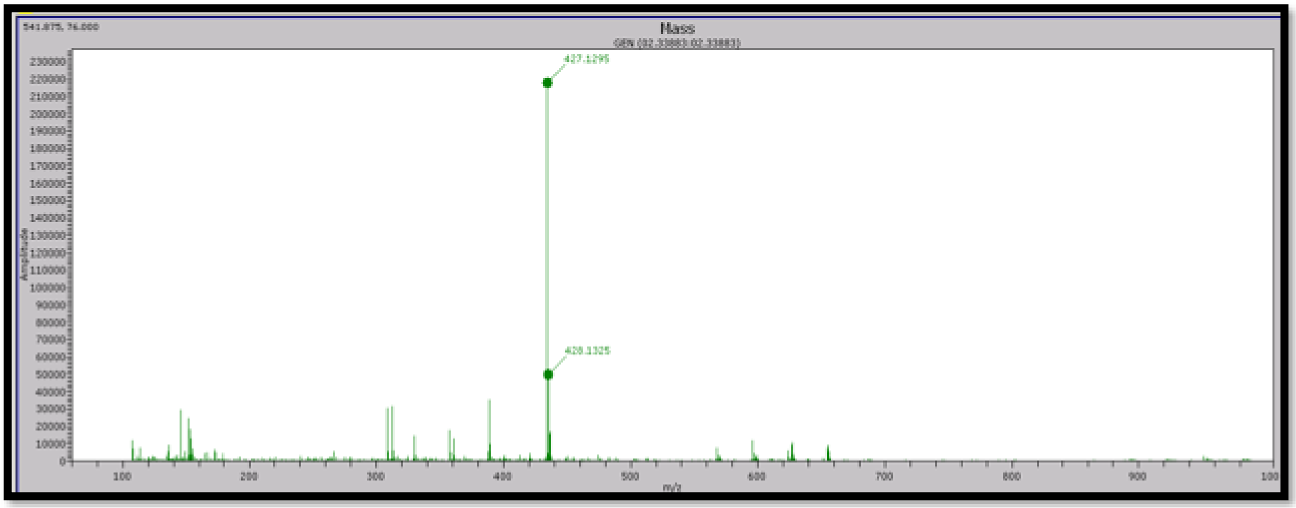

Mass
427.1295
428.1325
Amplitude
m/z

IR

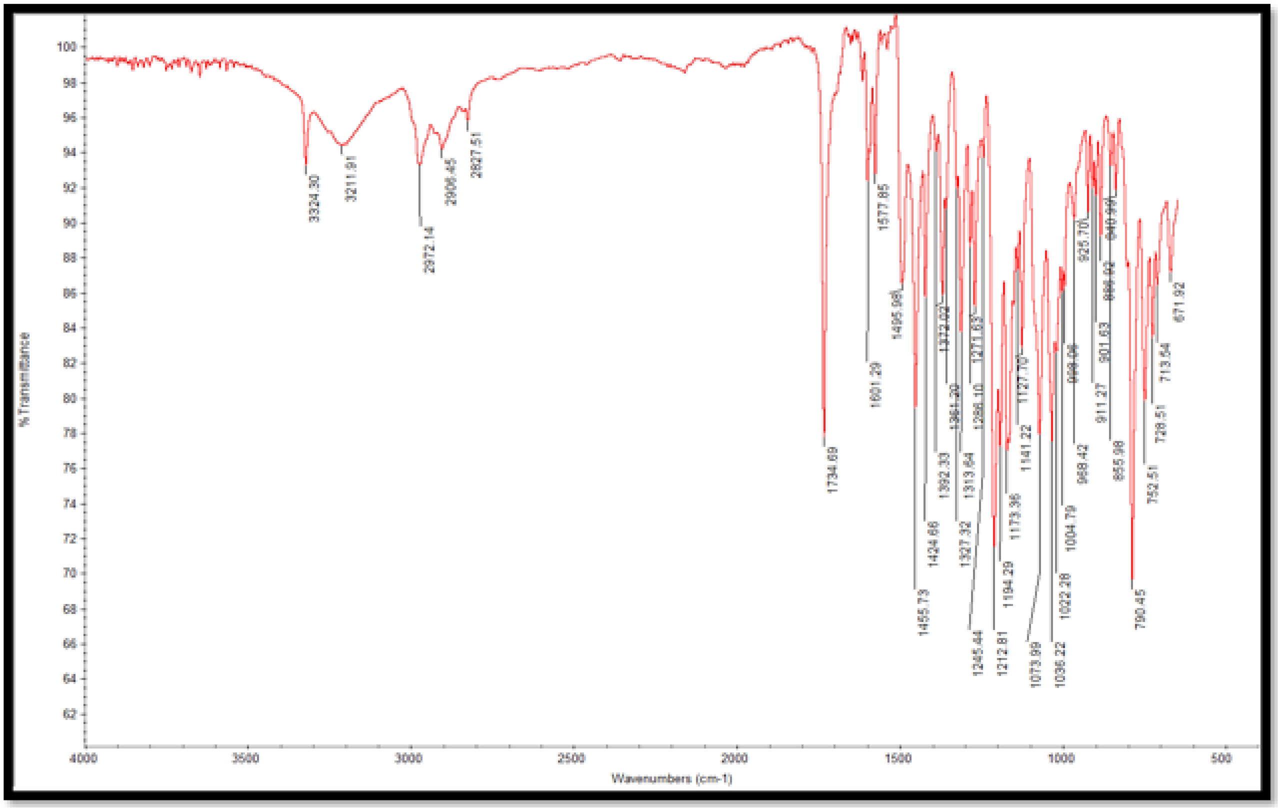
%Transmittance
Wavenumbers (cm-1)
3324.30
3211.91
2972.14
2906.45
2827.51
1734.69
1601.29
1577.85
1495.98
1455.73
1424.66
1392.33
1372.02
1361.20
1327.32
1313.64
1286.10
1271.63
1245.44
1212.81
1194.29
1173.36
1141.22
1127.70
1073.99
1036.22
1022.28
1004.79
968.42
959.08
935.70
911.27
901.63
888.02
855.98
840.99
790.45
752.51
728.51
713.54
671.92

HPLC

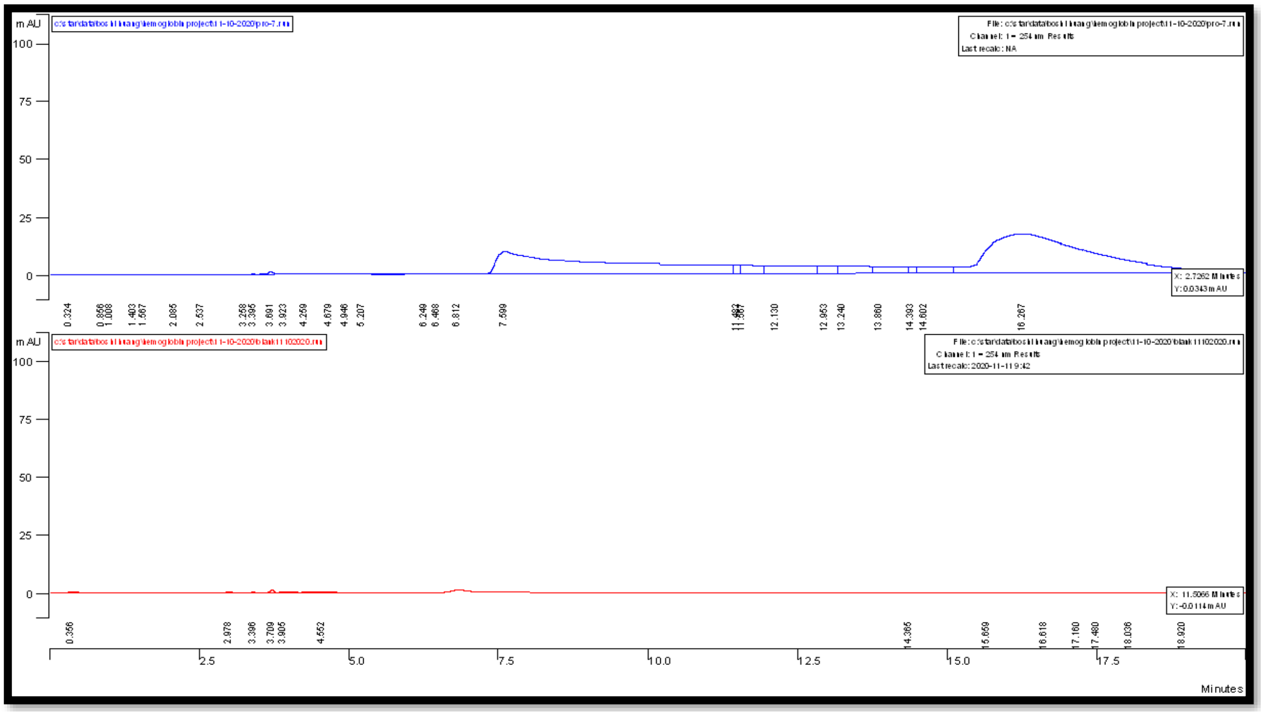

mAU
100
75
50
25
0
Channel: 1 = 254 nm Results
Last recalc: NA
X: 2.7262 Minutes
Y: 0.0343 mAU
0.324
0.856
1.008
1.403
1.567
2.085
2.537
3.258
3.395
3.691
3.923
4.259
4.679
4.946
5.207
6.249
6.468
6.812
7.599
12.130
12.953
13.240
13.860
14.393
14.602
16.267
mAU
100
75
50
25
0
Channel: 1 = 254 nm Results
Last recalc: 2020-11-11 9:42
X: 11.5066 Minutes
Y: -0.0114 mAU
0.356
2.978
3.396
3.709
3.905
4.552
14.365
15.659
16.618
17.160
17.480
18.036
18.920
2.5
5.0
7.5
10.0
12.5
15.0
17.5
Minutes
